# HBV Genotype B/C and Response to Lamivudine Therapy: A Systematic Review

**DOI:** 10.1155/2013/672614

**Published:** 2013-11-19

**Authors:** Xiu-Li Chen, Man Li, Xiao-Lan Zhang

**Affiliations:** ^1^Department of Gastroenterology, The Second Hospital of Hebei Medical University, Hebei Key Laboratory of Gastroenterology, Hebei Institute of Gastroenterology, No. 215 Heping West Road, Shijiazhuang 050000, China; ^2^Department of Epidemiology, School of Public Health, Hebei Medical University, Shijiazhuang, Hebei 050017, China

## Abstract

A number of nucleoside analogues such as lamivudine (LAM), actually used for the treatment of chronic hepatitis B, can suppress HBV DNA replication, improve transaminase level and liver histology, and enhance the rate of hepatitis B e antigen (HBeAg) clearance. The responses to LAM therapy involve HBeAg clearance and HBV DNA conversion of negative. However, the associations between HBV genotype B/C and response to LAM therapy remain ambiguous. The aim of this meta-analysis is to determine more precise estimations of the relationship. All the publications on the associations between HBV genotype B/C and response to LAM (HBeAg clearance and HBV DNA conversion of negative) through June 2013 were collected. Relative risk (RR) with 95% confidence intervals (95% CI) was calculated in fixed or random model, *I* ^2^ was calculated to examine heterogeneity, and funnel plots were plotted to examine small study effects with Stata 11 software. Overall, for HBeAg clearance and genotype B/C, the RR (95% CI) was 1.27 (0.94–1.71), while for HBV DNA conversion of negative and genotype B/C, the RR (95% CI) was 1.07 (0.98–1.17). HBV genotype B/C shows no significance associations with response to lamivudine therapy (HBeAg clearance and HBV DNA conversion of negative).

## 1. Introduction

Hepatitis B virus (HBV) infection is one of the major causes of hepatic cirrhosis and hepatocellular carcinoma in endemic areas [1]; the current study has determined that the different HBV genotypes have very important impacts on response to antiviral therapy [2, 3], in particular interferon treatment. LAM as a potent inhibitor of HBV reverse transcriptase activity is able to decrease serum HBV-DNA levels and improve the rate of HBeAg/anti-HBe seroconversion in patients with chronic hepatitis B (CHB). In regard to genotypes B and C, the Asian literature shows no difference in the antiviral (biochemical and virologic) response after 1 year of treatment with LAM 100 mg/daily orally in patients affected by HBeAg positive hepatitis [4–7]. These studies did not come into the conclusion and did not include some studies published recently. Data concerning the relevance of HBV genotypes in relation to response to LAM therapy are less clear. A meta-analysis is needed to combine all studies. Then in the present study, we will pool the data available and investigate the associations between genotypes B/C and response to LAM therapy susceptibility.

## 2. Methods

### 2.1. Testing Method

HBeAg and anti-HBe were tested by chemiluminescence enzyme immunoassay (CLEIA). The serum HBV DNA level in all the samples was quantified by the real-time PCR method. The detection limit of this assay was 100 copies/mL. The HBV genotype was determined by restriction fragment length polymorphism.

### 2.2. Study Selection

Several databases (PubMed, Web of Science, Science Direct, and CNKI Database) were searched through June 2012 for all publications on the association between genotype B/C and response to LAM therapy. The search terms were as follows: “genotype B” or “genotype C,” “LAM,” “HBeAg clearance,” and “DNA conversion of negative.” In addition, we also searched references of retrieved articles. 

### 2.3. Inclusion and Exclusion Criteria

The following inclusion criteria were used. Useful data were extracted with standardized data-extraction forms. Studies published in English and Chinese were eligible if they fulfilled the following criteria: (1) studies in the mentioned four databases with full text; (2) study data from published randomized controlled trials (RCTs); (3) the objects being CHB patients under lamivudine treatment with 100 mg daily, for 48 weeks, without other treatment measures taken at the same time; and (4) sufficient published data for estimating RR with 95% confidence interval (CI).

Studies were excluded if (1) study objects were patients with acute hepatitis B; (2) they were subset of a published article by the same authors; (3) studies present unclear data or obviously paradoxical data; (4) patients had received lamivudine therapy less than 12 months. Finally, we found 19 studies (16 articles), in which 9 studies were on the associations between genotype B/C and HBeAg clearance [8–16] and 10 studies between genotype B/C and HBV DNA conversion of negative [11, 12, 15, 17–23].

### 2.4. Evaluation Criteria

Primary end point of effectiveness of response was assessed after 24 weeks of treatment-free followup: HBeAg seroconversion (defined by the loss of HBeAg and the presence of anti-HBe antibody) and suppression of HBV DNA to levels below 100,000 copies per milliliter. Secondary end point of effectiveness of response assessed after 24 weeks included the combined response (HBeAg seroconversion, the normalization of alanine aminotransferase levels, and the suppression of HBV DNA levels to below 100,000 copies per milliliter), HBsAg seroconversion (defined by the loss of HBsAg and the presence of anti-HBs antibody), and the histologic response. A histologic response was defined as a reduction of at least two points in the modified Histologic Activity Index score as compared with the pretreatment score. 

### 2.5. Data Extraction

The following items were extracted from the literature: first author's last name, publication date, journal title, sample size, the status of HBeAg (positive or negative), the amount of serum HBV DNA (copies/mL), and HBV genotype. All the numerical values of HBV DNA copies were converted to logarithm. 

### 2.6. Statistical Analysis

Statistical analysis Review Manager 5.1 and Stata 11 software were used to perform meta-analysis. RR with 95% CIs were calculated in a fixed- or random-effect model to assess the strength of the association between genotype B/C and response to LAM therapy. Heterogeneity among studies was examined with *I* square (*I* ^2^) statistic [24] interpreted as the proportion of total variation contributed by between-study variation. If there was a statistical difference in terms of heterogeneity (*P* < 0.05), a random-effect model was selected to pool the data. A fixed-effect model, otherwise, was employed. Begg's funnel plot was used to examine small study effects. The method of Begg and Mazumdar [25] was used to calculate *P* for rank correlation and Egger's weighted regression method [26] to calculate *P* for bias. Throughout the paper, for HBeAg clearance (*t* = 0.18, *P* = 0.861) and for DNA conversion of negative (*t* = 2.28, *P* = 0.052), two-sided *P* values < 0.05 were considered as statistically significant.

## 3. Results

### 3.1. Search Results and Characteristics

**Figure 1:**
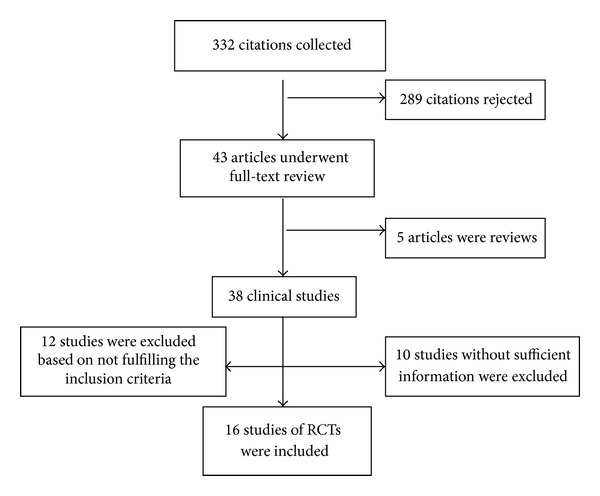
Flow diagram of the systematic literature research.

We identified 332 relevant literatures via electronic searches, the overlap studies were excluded, and finally 19 studies on randomized controlled trials (RCTs) were left for analysis which involved 3148 patients in total (who were included in LAM therapy groups) (Figure 1). All included trials had clearly stated inclusion and exclusion criteria. Of the 19 studies, 4 were published in Chinese [11–13, 17] and the others were published in English [8–10, 14–16, 18–23]. This meta-analysis identified 10 studies with a total number of 1860 cases for HBV DNA conversion of negative (and 9 studies with 1288 cases for HBeAg clearance), and the detailed information of included RCTs was summarized in Tables 1 and 2.

### 3.2. HBV DNA Conversion of Negative

**Figure 2:**
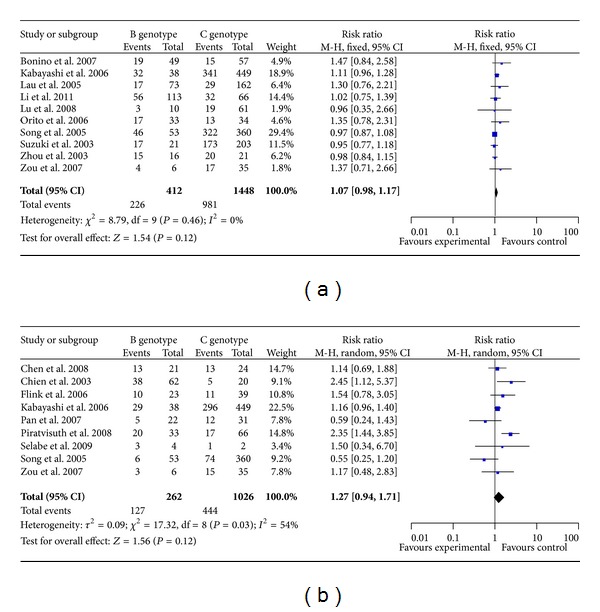
Forest plot for the associations of genotype B/C and HBV DNA conversion of negative (a) or HBeAg clearance (b) to lamivudine therapy.

**Figure 3:**
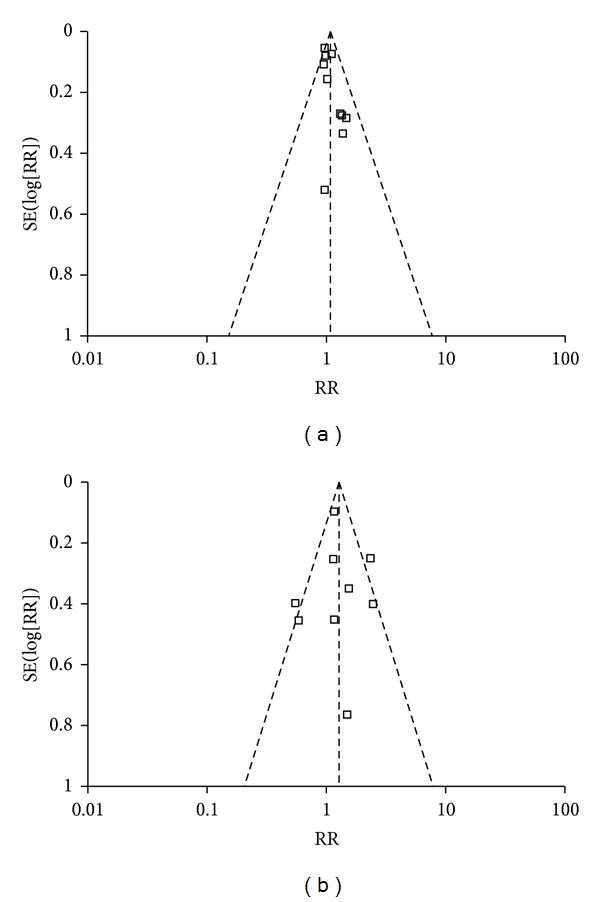
Begg's funnel plots for the associations of genotype B/C and HBV DNA conversion of negative (a) or HBeAg clearance (b) to lamivudine therapy.

According to chi-squared statistic and *I* ^2^, heterogeneity was assessed and was not found to be a concern. For HBV DNA conversion of negative, the overall RR (95% CI) associated with genotype B/C was 1.07 (0.98–1.17) (Figure 2(a)). Begg's funnel plot for the association between HBV DNA conversion of negative and genotype B/C was shown in Figure 3(a), in which *x*-axis is RR and *y*-axis is standard error (SE). No bias was observed for HBV DNA conversion of negative versus genotype B/C in Egger's test (*P* = 0.052). There was no heterogeneity in overall analysis (*I* ^2^ = 0, *P* = 0.46).

### 3.3. Hepatitis B e Antigen Clearance

According to chi-squared statistic and *I* ^2^, heterogeneity was assessed and *I* ^2^ showed moderate degree heterogeneity among studies (*I* ^2^ = 54%, *P* = 0.03). Of the nine analyzed trials, HBeAg clearance was observed in genotype B group as compared with that genotype C group, the overall RR (95% CIs) was 1.27 (0.94–1.71) (Figure 2(b)), and Begg's funnel plot for the association between HBeAg clearance and genotype B/C was shown in Figure 3(b), in which *x*-axis is RR and *y*-axis is standard error (SE). Smaller studies which lie in the right-hand side of the graph often have larger SEs. There was no evidence for bias using Egger's weighted regression method (*P* = 0.861). 

## 4. Discussion

Chronic HBV infection is a dynamic state of interactions between the virus, hepatocytes and the host immune response [27–29]. HBV genotypes (A to H) can be classified based on comparison of complete HBV genomes and in accordance with the criterion of 8% or more differences in the complete nucleotide sequence of the viral genome [2, 3]. Genotypes B and C are common in the majority of Asian countries. In fact, with regard to HBV genotypes B and C, which are prevalent in Asia, genotype C has been shown to be more frequently found in severe liver disease and in hepatocellular carcinoma, whereas genotype B is associated with faster HBeAg/anti-HBe seroconversion during acute hepatitis [30–32]. At present, it is not clear whether viral genotype is a predictor of treatment response in chronic hepatitis B, as it is in chronic hepatitis C. Responses to all genotypes, nucleoside or nucleotide analogues are generally consistent among all genotypes. To our knowledge, no systematic review has been previously published on the association between genotype B/C and response to LAM therapy.

In this meta-analysis, all informed consent of patients were obtained. CHB patients received lamivudine, 100 mg daily, for 48 weeks, without other treatment measures taken at the same time. The patients with normal ALT levels (40 IU/L or less) before commencement of the treatment were excluded. All patients were positive for HBsAg for more than 6 months and negative for both HCV and anti-HIV. All patients were tested by ultrasonography or CT scan to rule out decompensated cirrhosis or hepatocellular carcinoma.

 Response rates were calculated for all patients according to the intention-to-treat principle. For the patients, who received at least one dose of study drug, including both HBeAg and HBV DNA groups, the response rate of HBV DNA to lamivudine therapy about HBV genotype B/C was defined as the ratio of the HBV DNA conversion of negative, and the response rate of HBeAg to lamivudine therapy about HBV genotype B/C was defined as the ratio of the HBeAg clearance. HBeAg clearance was evaluated in 1288 patients with CHB who received LAM therapy between patients with genotype B and genotype C. Results from currently available studies suggest that HBeAg clearance was observed in genotype B group as compared with genotype C; the overall RR (95% CI) was 1.27 (0.94–1.71). The present study showed that genotype B/C was negatively associated with HBeAg clearance to LAM therapy. While a retrospective-prospective study suggested that genotype B, age less than 36 years, and additional LAM treatment over 8 months after HBeAg seroconversion showed a sustained HBeAg response up to 80% (*P* < 0.005) [16], this study indicates that age and additional treatment may be the major determinants [33–35]. 

A total of 10 studies were included in following analysis. The results showed no differences in terms of HBV DNA conversion of negative at week 52, between patients with genotype B and genotype C, which agree with the outcomes of previous studies [19–23, 36–38]; the overall RR (95% CIs) was 1.07 (0.98–1.17). No association was found between genotype B/C and HBV DNA conversion of negative to LAM therapy. Funnel plots and two formal statistical methods were used to detect bias. 

It should be noted that some limitations existed in this meta-analysis. It is well known to us that many factors influence the outcomes to LAM therapy, including country of origin, ethnicity, age, gender, severity of liver disease, baseline of HBV DNA, numbers of cases and controls, and genotyping method, which may introduce errors in analysis.

In conclusion, this meta-analysis indicated that genotype B/C was not associated with response to LAM therapy (HBeAg seroclearance and HBV DNA conversion of negative). Further mechanism researches are required to clarify. Large-scale population studies in multicountries are also necessary to evaluate the influence of HBV genotypes in hepatitis B progression and antiviral treatment.

## Figures and Tables

**Table 1:** Characteristics of HBV DNA studies included in the meta-analysis.

Author	Year	*N*	Age (years)	Gender (M%)	Genotype (B/C)	Severity of liver disease	HBVDNA (log copies/mL)
Lu et al. [17]	2008	71	32 ± 9.0	83	10/61	CHB	7.7 ± 0.8
Li et al. [18]	2011	179	35.4 ± 10.3	80	113/66	CHB	9.4 ± 1.3
Orito et al. [19]	2006	67	41.5 ± 12.2	67	33/34	CHB	10.7 ± 1.1
Zou et al. [11]	2007	41	32.1 ± 9.6	85	6/35	CHB	5.8 ± 1.2
Song et al. [12]	2005	413	—	—	53/360	CHB	—
Bonino et al. [20]	2007	106	37.9 ± 10.6	82	49/57	CHB	8.9 ± 1.1
Lau et al. [21]	2005	235	31.6 ± 9.7	91	73/162	CHB	10.1 ± 2.0
Kobayashi et al. [15]	2006	487	45.1 ± 13.1	86	38/449	CHB	7.2 ± 0.0
Suzuki et al. [22]	2003	224	46 ± 12.1	—	21/203	CHB	9.1 ± 1.6
Zhou et al. [23]	2003	37	—	—	16/21	CHB	8.3 ± 1.2

*N*: numbers of patients; CHB: chronic hepatitis B.

**Table 2:** Characteristics of HBeAg studies included in the meta-analysis.

Author	Year	*N*	Age	Sex	Genotype B/C	Severity of liver disease	HBeAg
Zou et al. [11]	2007	41	32.1 ± 9.6	85	6/35	CHB	Positive
Song et al. [12]	2005	413	—	—	53/360	CHB	Positive
Kobayashi et al. [15]	2006	487	45.1 ± 13.1	81	38/449	CHB	Positive
Flink et al. [8]	2006	62	34 ± 9.0	69	23/39	CHB	Positive
Pan et al. [9]	2007	53	53 ± 12	57	22/31	CHB	Positive
Piratvisuth et al. [10]	2008	99	—	—	33/66	CHB	Positive
Chen et al. [13]	2008	45	30 ± 10.7	76	21/24	CHB	Positive
Selabe et al. [14]	2009	6	45 ± 12.6	83	4/2	CHB	Positive
Chien et al. [16]	2003	82	33.8 ± 11.1	77	62/20	CHB	Positive

*N*: numbers of patients; CHB: chronic hepatitis B.
